# A HIF-1*α*-driven feed-forward loop augments HIF signalling in Hep3B cells by upregulation of ARNT

**DOI:** 10.1038/cddis.2016.187

**Published:** 2016-06-30

**Authors:** M Mandl, M-K Lieberum, R Depping

**Affiliations:** 1 Institute of Physiology, Center for Structural and Cell Biology in Medicine, University of Luebeck, Ratzeburger Allee 160, Luebeck 23562, Germany; 2 Klinik fuer Strahlentherapie, Universitaetsklinikum Schleswig-Holstein, Campus Luebeck, Ratzeburger Allee 160, Luebeck 23538, Germany

## Abstract

Oxygen-deprived (hypoxic) areas are commonly found within neoplasms caused by excessive cell proliferation. The transcription factor Aryl hydrocarbon receptor nuclear translocator (ARNT) is part of the hypoxia-inducible factor (HIF) pathway, which mediates adaptive responses to ensure cellular survival under hypoxic conditions. HIF signalling leads to metabolic alterations, invasion/metastasis and the induction of angiogenesis in addition to radio-chemoresistance of tumour cells. Activation of the HIF pathway is based on the abundance of HIF-*α* subunits, which are regulated in an oxygen-dependent manner and form transcriptional active complexes with ARNT or ARNT2 (also referred as HIF-1*β* and HIF-2*β,* respectively). ARNT is considered to be unaffected by hypoxia but certain cell lines, including Hep3B cells, are capable to elevate this transcription factor in response to oxygen deprivation, which implies an advantage. Therefore, the aim of this study was to elucidate the mechanism of hypoxia-dependent ARNT upregulation and to determine implications on HIF signalling. Gene silencing and overexpression techniques were used to alter the expression pattern of HIF transcription factors under normoxic and hypoxic conditions. qRT-PCR and western blotting were performed to measure gene and protein expression, respectively. HIF activity was determined by reporter gene assays. The results revealed a HIF-1*α*-dependent mechanism leading to ARNT upregulation in hypoxia. Forced expression of ARNT increased reporter activity under normoxic and hypoxic conditions. In conclusion, these findings indicate a novel feed-forward loop and suggest that ARNT might be a limiting factor. Augmented HIF signalling in terms of elevated target gene expression might be advantageous for tumour cells.

A heterogeneous oxygenation is a characteristic attribute of solid tumours. Oxygen-deprived, that is hypoxic, areas are commonly found within neoplasms owing to uncontrolled cell proliferation.^1, 2, 3^ In general, tumour hypoxia is considered as a negative prognostic marker associated with resistance to radio-chemotherapy and poor patient outcome.^1, 2, 4^

To ensure the survival of tumour cells, these are forced to initiate adaptive responses to an insufficient oxygen supply.^2^ This is mediated by the hypoxia-inducible factor (HIF) pathway, which triggers a number of cellular alterations affecting proliferation, metabolism and invasion/metastasis. Furthermore, the induction of angiogenesis is a HIF-dependent mediated process in order to increase the available oxygen concentration.^2^ Owing to its tumour-promoting capacity, inhibition of the HIF pathway by several strategies is regarded as a treatment option in cancer therapy.^2, 4, 5, 6, 7, 8, 9, 10^

The HIF proteins belong to the Per-ARNT-Sim family of transcription factors and act as heterodimers.^1, 4, 11^ Among them, three HIF-*α* subunits (HIF-1*α*, HIF-2*α*, HIF-3*α*) and two beta subunits have been described.^4^ The alpha subunits are regulated in an oxygen-dependent manner but differ in their expression pattern and tumourigenic potential.^4^ HIF-1*α* is expressed ubiquitously, whereas HIF-2*α* expression is more restricted to specific cell types including hepatocytes.^1, 4^ Different HIF-3*α* splice variants exist, which are able to activate or repress HIF signalling depending on the cellular context.^4, 12, 13^

The beta subunits Aryl hydrocarbon receptor nuclear translocator (ARNT) and ARNT2, also designated as HIF-1*β* and HIF-2*β*, respectively, serve as obligatory binding partners for HIF-*α* subunits in order to form functional complexes.^4^ ARNT is present in all tissues and regarded to be constitutively expressed, meaning to be unaffected by oxygen tension (despite the name HIF-1*β*).^1, 4, 14^ In addition, it is assumed that ARNT is available in excess inside the cell.^15^ However, there is accumulating evidence that certain cell lines are capable to upregulate ARNT in hypoxia,^16, 17, 18^ which implies a cellular advantage.^19^ This attribute was first described in Hep3B hepatocellular carcinoma cells by *Wang et al.*^20^ in 1995. ARNT2 is an ARNT homologue^21^ and expressed in a limited number of cell types,^4^ whereas many functions of this protein remain unknown.^22^ For instance, a recent study demonstrated an inhibitory role of ARNT2 in hepatocellular carcinoma progression.^21^

Under normoxic conditions, HIF-*α* is hydroxylated at two conserved proline residues by prolyl hydroxylase domain enzymes. Subsequently, this post-translational modification is recognised by the von Hippel-Lindau tumour suppressor protein, which targets the alpha subunits for proteasomal degradation.^4, 5^ In contrast, oxygen deprivation prevents prolyl hydroxylase domain activity and leads to HIF-*α* accumulation followed by nuclear translocation.^5^ Within the nucleus, HIF-*α* dimerises with ARNT (or ARNT2) and binds to hypoxia-responsive elements (HRE) commonly found within regulatory sequences of HIF transcribed genes.^4, 5^ HIF-1 and HIF-2, which are composed of either HIF-1*α* or HIF-2*α* and ARNT, respectively, are the main players within this pathway.^4, 23^ The expression of specific target genes is initiated in conjunction with cofactors such as CBP/p300.^4^ The plethora of HIF regulated genes encode for growth factors (e.g., vascular endothelial growth factor),^2, 4^ transporters (e.g., glucose transporter 1),^2, 4^ enzymes (e.g., lactate dehydrogenase),^2, 4^ transcription factors (e.g., TWIST1, Oct4)^14, 24^ or microRNAs^14^ among others.^14, 24^

Recently, we demonstrated that an elevated ARNT expression level confers radioresistance in tumour cells (i.e., in Hep3B), which renders the regulation of this transcription factor clinically important.^19^ Furthermore, other studies revealed a major contribution of ARNT in hepatocellular carcinoma progression^25^ and point towards ARNT as a potential drug target regarding this malignancy.^26^

Therefore, the objective of the present study was to elucidate the mechanism of hypoxia-dependent ARNT upregulation in Hep3B cells.

The results revealed a non-canonical regulatory relationship between HIF-1*α* and ARNT. Elevation of ARNT in hypoxia was mediated by a HIF-1*α*-dependent mechanism. Additional experiments showed that ARNT overexpression was sufficient to stabilise HIF-1*α* and to activate HRE-driven reporter gene expression in normoxia. Moreover, reporter activity was further increased in hypoxic Hep3B cells transiently transfected with the ARNT expression vector.

In conclusion, the presented data reveal that an elevated amount of ARNT augments HIF signalling in Hep3B cells and implies that ARNT is a limiting factor in this model.

## Results

### ARNT but not ARNT2 is elevated in hypoxic Hep3B cells

**Figure 1:**
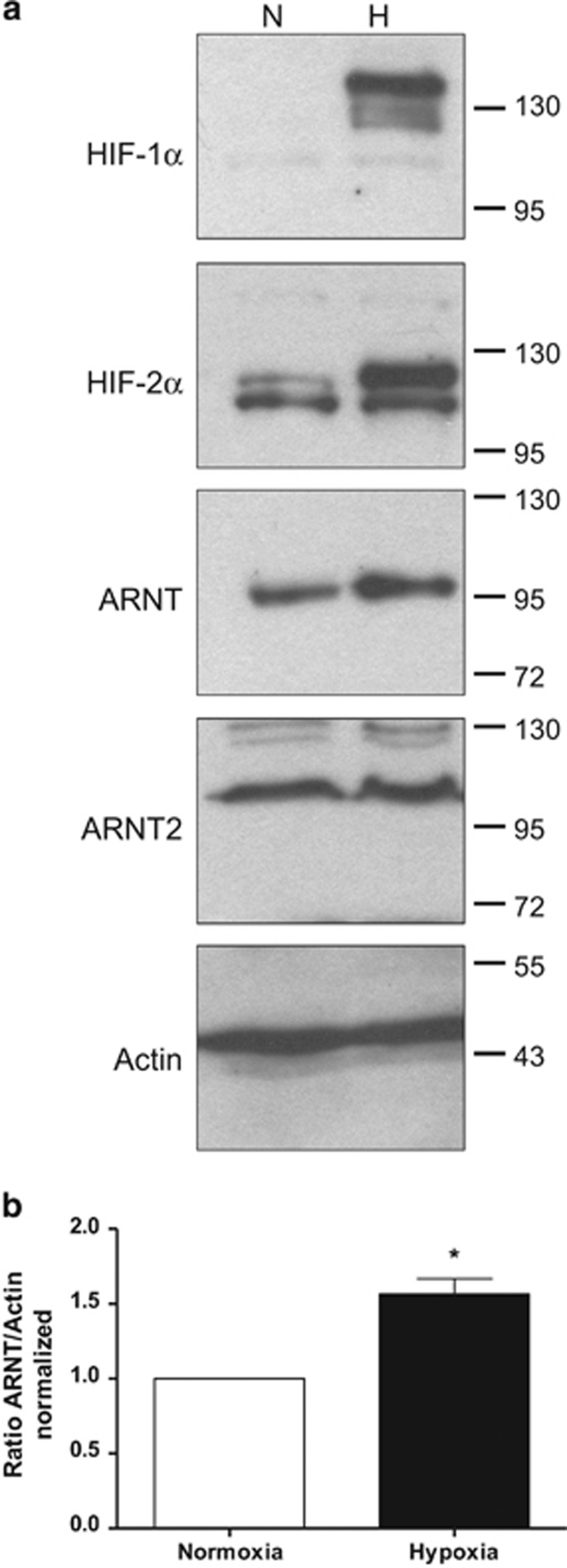
Western blot analysis of normoxic (N) or hypoxic (H) Hep3B cells. (**a**) Representative result of *n*=3 independent experiments. Protein masses are given in kDa. (**b**) Densitometry of ARNT protein levels. The ratio of ARNT/Actin was calculated and normalised to normoxic control cells. Values are presented as mean±S.E.M. of *n*=3 independent experiments. Statistical analysis was done using the one-sample *t*-test

A previous study published in 1995 by *Wang et al.*^20^ demonstrated the capability of Hep3B cells to upregulate ARNT in hypoxia. This cellular trait was also confirmed by *Wolff et al.*^18^ who showed that the induction of ARNT was more pronounced in an hypoxic environment of 3% O_2_ as compared with 1% O_2_. To investigate whether the protein level of the ARNT homologue ARNT2 might be affected by low-oxygen tension as well, Hep3B cells were exposed to 3% O_2_ or cultured in normoxia for 8h followed by western blot analysis. As shown in Figure 1, HIF-1*α* and HIF-2*α* were induced in hypoxic cells as expected. In addition, ARNT was upregulated in cells cultured under low-oxygen tension. By contrast, ARNT2 protein levels remained unaffected owing to hypoxia.

**Figure 2:**
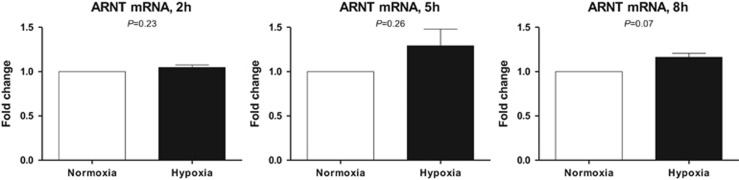
qRT-PCR analysis of ARNT mRNA expression in Hep3B cells cultured in normoxia or hypoxia for 2, 5 and 8h, respectively. ARNT mRNA was measured using the ΔΔC_t_ method relative to B2M expression (endogenous control). ARNT mRNA expression was normalised to appropriate normoxic controls. Each graph represents the results of *n*=3 independent experiments. Values are given as mean±S.E.M. Statistical analysis was conducted using the one-sample *t*-test. Appropriate *P*-values are indicated

To test whether the inducibility of ARNT originates from an altered mRNA level, qRT-PCR analysis was performed. ARNT mRNA levels of Hep3B cells exposed to hypoxia for 2, 5 and 8h were measured and compared with appropriate normoxic controls. Under these conditions, ARNT mRNA seemed to be slightly elevated in hypoxic cells after 5h and 8h, respectively (Figure 2).

Taken together, these findings confirm previous reports and establish Hep3B cells as a suitable model to study the hypoxia-dependent upregulation of ARNT.

### Hypoxia-dependent upregulation of ARNT is mediated by HIF-1*α*

**Figure 3:**
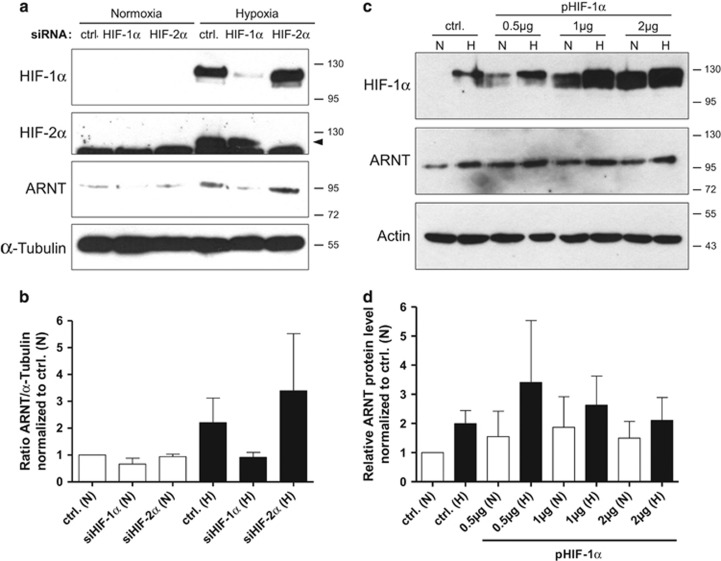
Knockdown and overexpression studies in Hep3B cells. (**a**) Western blot analysis of siRNA-transfected cells exposed to normoxia or hypoxia for 8 h. The Arrow indicates the specific signal for HIF-2*α*. Protein masses are given in kDa. ctrl.: control siRNA-transfected cells. Representative result of *n*=3 independent experiments. (**b**) Densitometric analysis of ARNT protein expression in knockdown cells (corresponding to **a**). The ARNT/*α*-Tubulin ratio was calculated and normalised to normoxic control cells. Values are presented as mean±S.E.M. of *n*=3 independent experiments. (**c**) Western blot analysis of transiently transfected cells using increasing amounts of the HIF-1*α* expression vector (pHIF-1*α*) in conjunction with normoxic (N) and hypoxic (H) conditions. Protein masses are given in kDa. ctrl.: control transfected cells (pcDNA3, 2*μ*g/well). Representative result of *n*=3 independent experiments. (**d**) Densitometric analysis of ARNT protein expression in HIF-1*α-*overexpressing cells (corresponding to **c**). The ARNT signal was set in ratio to the appropriate loading control and normalised to normoxic control cells. Values are presented as mean±S.E.M. of *n*=3 independent experiments

A recent study demonstrated that HIF-1*α* enables the inducibility of ARNT under hypoxic conditions in a human melanoma cell line.^17^ In order to test whether this non-canonical regulatory relationship applies for Hep3B cells too, knockdown experiments were conducted. As shown in Figure 3a and b, HIF-1*α*, HIF-2*α* and ARNT were elevated in control siRNA-transfected cells exposed to hypoxia. Transfection with siRNA against HIF-1*α* depleted the protein in hypoxic Hep3B cells and prevented the upregulation of ARNT. In contrast, silencing of HIF-2*α* diminished this subunit under low-oxygen tension as expected, but did not inhibit the inducibility of ARNT.

Next, overexpression experiments were performed in order to confirm this finding. Hep3B cells were transiently transfected either with increasing amounts of a HIF-1*α* expression vector or the corresponding empty plasmid and exposed to hypoxia or maintained under normoxic conditions (Figures 3c and d). As expected, both HIF-1*α* and ARNT were induced in hypoxic control cells compared with normoxic counterparts. Interestingly, overexpression of HIF-1*α* seemed to be sufficient to elevate ARNT in normoxia. ARNT was further increased in HIF-1*α* overexpressing cells exposed to hypoxia. This finding indicates *de novo* synthesis of ARNT and/or the possibility of reduced turnover due to HIF-1*α* heterodimerization.

In summary, these results demonstrate a HIF-1*α*-dependent mechanism leading to ARNT upregulation in hypoxia.

### HIF-1*α* does not prevent ARNT from degradation

**Figure 4:**
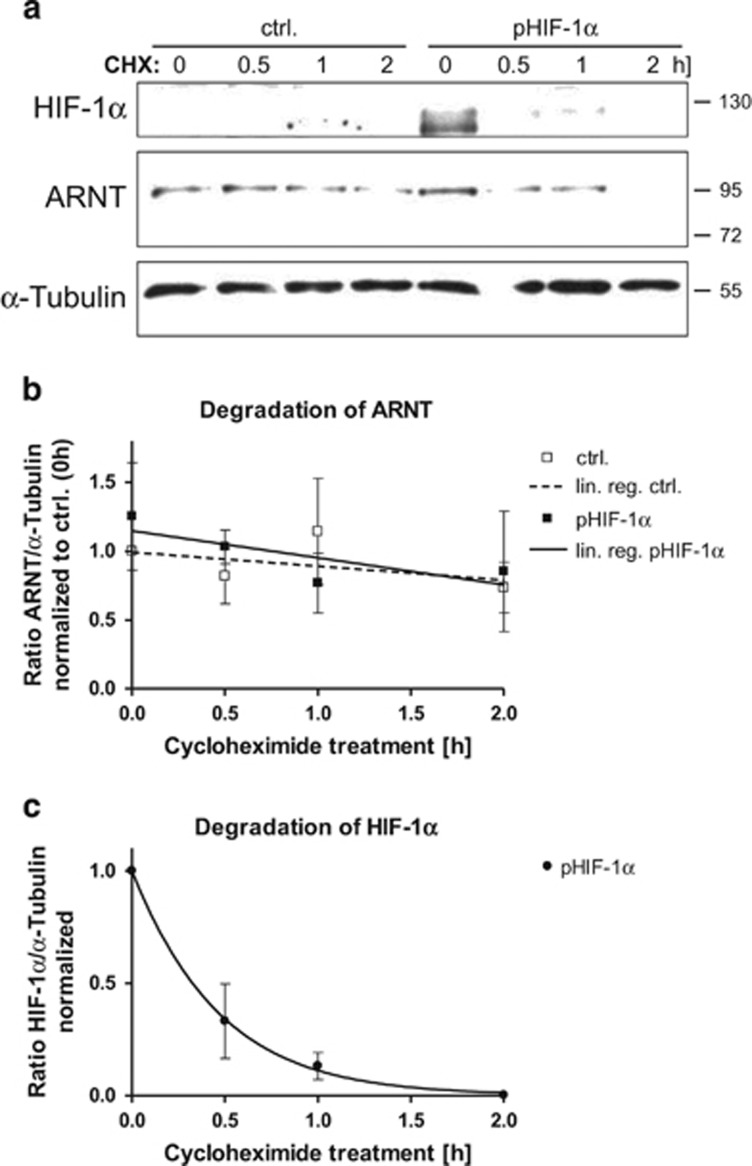
Cycloheximide (CHX) chase experiments in transfected Hep3B cells in normoxia. (**a**) Western blot analysis of HIF-1*α* and ARNT degradation after CHX treatment (100 *μ*M) in control (ctrl.) and pHIF-1*α-*transfected cells. Protein masses are given in kDa. Representative result of *n*=3 independent experiments. (**b**) Quantitation of ARNT degradation. Values represent mean±S.E.M. of *n*=3 (except ctrl. 1 h: *n*=2) independent experiments; lin. reg.: linear regression. (**c**) Quantitation of HIF-1*α* degradation in pHIF-1*α*-transfected cells. Values represent mean±S.E.M. of *n*=3 independent experiments

To address the issue whether HIF-1*α* might affect ARNT degradation in Hep3B cells, cycloheximide (CHX) chase experiments were performed. For this purpose cells were transiently transfected either with the HIF-1*α* expression vector or the empty control plasmid and treated with CHX for various time points in normoxia. Subsequently, degradation of HIF-1*α* and ARNT was detected by western blotting (Figure 4a) and quantified. As shown in Figure 4b, overexpression of HIF-1*α* elevated ARNT under normoxic conditions compared with empty vector-transfected cells. Decline of ARNT over time owing to CHX treatment was slow in both control and HIF-1*α-*overexpressing cells. As expected, HIF-1*α* did not delay ARNT degradation (slope: ctrl.: −0.1013±0.1254 *versus* pHIF-1*α*: −0.1956±0.1990). In contrast, turnover of HIF-1*α* occurred rapidly by the onset of CHX treatment and followed a different kinetics (Figure 4c). These findings argue for HIF-1*α* mediated *de novo* synthesis of ARNT in Hep3B cells.

### Hypoxia-dependent upregulation of ARNT depends on RNA synthesis

**Figure 5:**
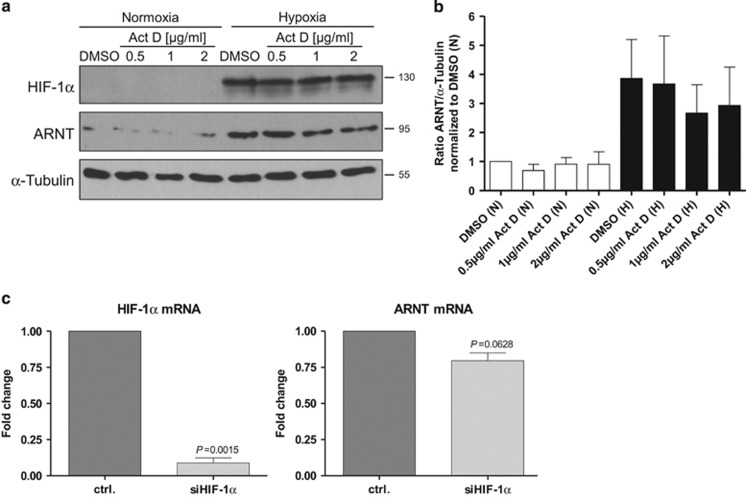
Effects on transcription. (**a**) Treatment of Hep3B cells with Actinomycin D (Act D). Cells were incubated with increasing concentrations of Act D in normoxia (N) and hypoxia (H, 3% O_2_) for 8 h as indicated. DMSO at the highest concentration (0.1 v/v %) was used as vehicle control. HIF-1*α* and ARNT protein levels were determined by western blot analysis. Representative result of *n*=3 independent experiments. (**b**) Densitometry of ARNT protein levels (corresponding to a). The ratio of ARNT/*α*-Tubulin was calculated and normalised to normoxic vehicle-treated control cells. Values are given as mean±S.E.M. of *n*=3 independent experiments. (**c**) Knockdown of HIF-1*α* in hypoxic Hep3B cells. Cells were transfected either with control (ctrl.) or HIF-1*α* siRNA (siHIF-1*α*) and exposed to 3% O_2_ for 5 h. HIF-1*α* and ARNT mRNA expression were measured by qRT-PCR as described in materials and methods. Normalised expression levels are shown. Values are presented as mean±S.E.M. of *n*=3 independent experiments. Statistical comparison was performed using the one-sample *t*-test. Appropriate *P-*values are indicated

The data presented above indicate that hypoxia-dependent ARNT upregulation is achieved by *de novo* synthesis in Hep3B cells. Thus, the inducibility of ARNT should be sensitive to Actinomycin D (Act D) treatment. To investigate this issue, Hep3B cells were incubated with increasing concentrations of Act D under normoxic and hypoxic conditions for 8h. Afterwards western blot analysis was used in order to determine HIF-1*α* and ARNT protein levels. As anticipated, HIF-1*α* accumulated in hypoxia compared with normoxic counterparts. In addition, ARNT was elevated owing to oxygen deprivation (Figure 5a and Supplementary Figure S1). Act D treatment diminished the upregulation of ARNT under hypoxic conditions in a dose-dependent manner (Figure 5a and b and Supplementary Figure S1). This observation further supports previous results and demonstrates that hypoxia-dependent elevation of ARNT relies predominantly on RNA synthesis.

To strengthen this issue, HIF-1*α* was knocked down and ARNT mRNA expression was determined in hypoxic Hep3B cells. As presented in Figure 5c, silencing of HIF-1*α* caused a decline of the ARNT mRNA level. This finding indicates that HIF-1*α* regulates the expression of its binding partner ARNT on the transcriptional level.

### Overexpression of ARNT leads to HIF-1*α* accumulation in normoxia

**Figure 6:**
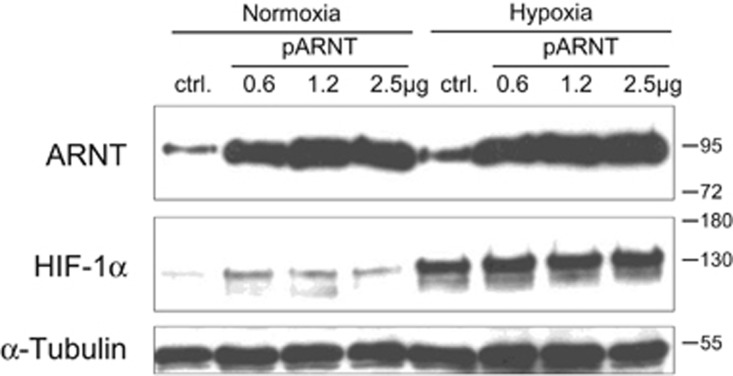
Overexpression of ARNT in Hep3B cells. ctrl.: control (pcDNA3, 2.5 *μ*g/well) transfected cells. Representative result of *n*=3 independent experiments

To test whether an elevated ARNT level might affect HIF-1*α* stability as previously demonstrated by *Isaacs et al.*,^27^ Hep3B cells were transiently transfected with increasing concentrations of an ARNT expression vector or the empty control plasmid. Subsequently, cells were maintained in normoxia or hypoxia for 8h and subjected to western blot analysis. As shown in Figure 6, ARNT was elevated in hypoxic control cells as compared with normoxic counterparts. ARNT overexpression was sufficient to upregulate its binding partner HIF-1*α* in normoxia. Taken together, these results demonstrate that an increased ARNT expression elevates the amount of HIF-1*α* in Hep3B cells.

### An elevated ARNT level is sufficient to initiate HIF signalling in normoxia

**Figure 7:**
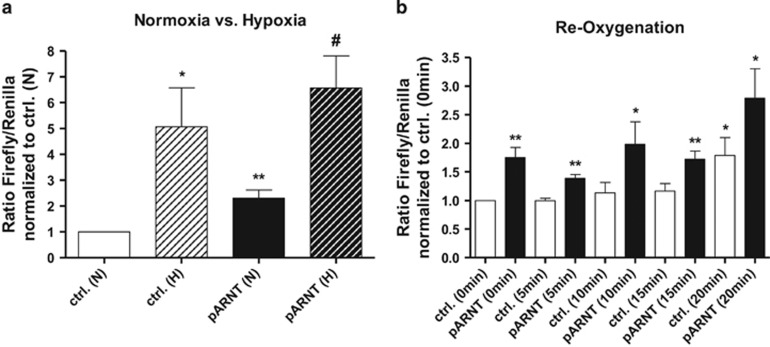
Luciferase assays. (**a**) Reporter gene expression of control (ctrl.) or pARNT-transfected cells incubated in normoxia (N) or hypoxia (H) for 8 h. Values represent the mean±S.E.M. of *n*=4 independent experiments. Statistical comparison of two treatment groups (*versus ctrl. (N) or #*versus* pARNT (N)) was done using the unpaired *t*-test. (**b**) Reporter gene expression of control (ctrl.) or pARNT-transfected cells exposed to hypoxia for 8h followed by re-oxygenation for indicated time points. Values represent the mean±S.E.M. of *n*=4 independent experiments. Statistical comparison of treatment groups *versus* ctrl. (0 min) was achieved using the unpaired *t*-test

The activity of the HIF pathway is tightly regulated by the accumulation of HIF-*α* subunits under hypoxic conditions.^4, 28^ Therefore, it was hypothesised that accumulation of HIF-1*α* due to ARNT overexpression might lead to HIF transactivation. In order to test this assumption, reporter gene assays using a HRE-reporter construct were conducted. Interestingly, cells transiently transfected with the ARNT expression vector showed a significant induction of HRE-reporter activity in normoxia as compared with the appropriate control (Figure 7a). In addition, overexpression of ARNT further increased the *Firefly/Renilla* luciferase ratio in hypoxia compared with cells harbouring the empty plasmid. These findings demonstrate that elevation of ARNT is sufficient to activate HIF signalling under normoxic conditions and amplifies the response in hypoxia indicating to be a limiting factor in this cell line.

In order to evaluate the endurance of this effect after termination of hypoxia, re-oxygenation experiments were performed. Thus, Hep3B cells were transfected as described above and subjected to hypoxia for 8h followed by normoxia for indicated time points (Figure 7b). Interestingly, the *Firefly/Renilla* luciferase ratio significantly increased after 20 min in vector-transfected control cells. A similar effect was observed in ARNT-overexpressing cells. Noteworthily, HRE-reporter activity was increased in pARNT-transfected cells compared with appropriate controls at all time points tested. These measurements confirm the findings from above and indicate augmented HIF signalling in ARNT-overexpressing cells even after short-term re-oxygenation.

### ARNT is degraded after re-oxygenation

**Figure 8:**
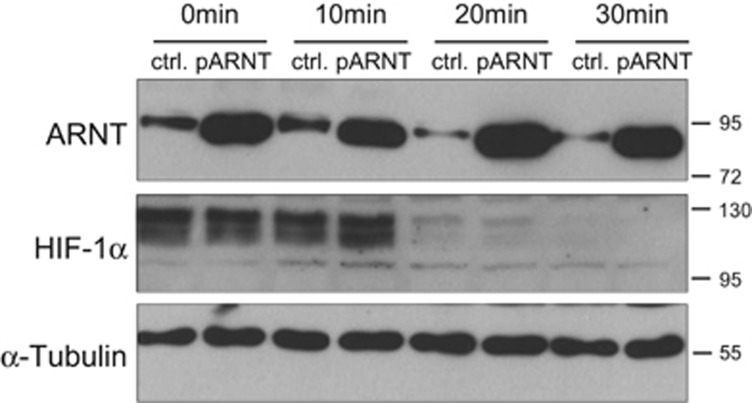
Re-oxygenation of control and ARNT-overexpressing Hep3B cells. Transiently transfected control (ctrl.) or ARNT-overexpressing (pARNT) cells were incubated in hypoxia (3% O_2_) for 8 h and subsequently exposed to normoxia for indicated time points followed by western blot analysis. Representative result of *n*=3 independent experiments

ARNT-induced HIF-1*α* accumulation in ARNT-overexpressing cells (Figure 6) might be the cause for the observed prolonged HRE-reporter activity after re-oxygenation (Figure 7b). To test this possibility in addition to separate hypoxic and ARNT-mediated effects on HIF-1*α*, re-oxygenation experiments were performed as outlined above using control or pARNT-transfected Hep3B cells. Subsequently, protein levels of ARNT and HIF-1*α* at indicated time points were determined by western blot analysis (Figure 8). Interestingly, the endogenous ARNT level declined after re-oxygenation in control cells, whereas ARNT was stably overexpressed in appropriate cells. As expected, HIF-1*α* was degraded in a time-dependent manner in normoxia. Overexpression of ARNT had no pronounced effect on HIF-1*α* turnover under these conditions. In summary, these findings imply that hypoxia-dependent ARNT upregulation is a temporal mechanism and reversed by re-oxygenation.

## Discussion

The capability of Hep3B human hepatocellular carcinoma cells to elevate the transcription factor ARNT in response to hypoxia was first described by *Wang et al.*^20^ in 1995. The data presented in our study are in line with this report^20^ although different experimental conditions were used. In our model the elevation of ARNT was more pronounced on protein rather than mRNA level (Figure 1 and Figure 2), which might also indicate a post-transcriptional regulation. Indeed, we recently revealed cell-specific differences between ARNT mRNA and protein expression in other cell lines.^19^

According to the general point of view ARNT is constitutively expressed.^4^ This means that neither ARNT mRNA nor protein level is affected by environmental conditions such as hypoxia. However, until now certain cell lines have been described, which harbour the capability to upregulate ARNT in hypoxia.^16^ In addition, this exceptional trait implies a cellular benefit during tumourigenesis (reviewed in ref. 16).

Since the aforementioned major finding described by *Wang et al.*^20^ only a limited number of studies focussed on the regulation of ARNT under hypoxic conditions.^16^ Recently, *Wolff et al.*^18^ demonstrated that ARNT is influenced by hypoxia and hypoxia-mimetics in a number of cell lines. This observation implies that an active hypoxic regulation of ARNT is not restricted to a specific tumour entity.^18^ One main finding of our current study is the non-canonical regulatory relationship between HIF-1*α* and ARNT. The requirement of HIF-1*α* to elevate ARNT under hypoxic conditions in Hep3B cells strongly parallels a mechanism already demonstrated in 518A2 human melanoma cells.^17^ In this cell line the same regulatory relationship between HIF-1*α* and its binding partner ARNT was described.^17^ Whether this cellular trait relies on the same mechanism or is the outcome of different alterations in both cell models is unclear. Nevertheless, it fits into the concept that tumour cells might acquire the capability to elevate ARNT in response to hypoxia in order to obtain a survival advantage.

Therefore, the question comes up how HIF-1*α* might regulate the expression of ARNT. Direct HIF target genes are characterised by the presence of HRE sequences. In addition, a secondary hypoxic response exists. This is mediated by transcription factors, chromatin modifiers and microRNAs itself encoded by HIF regulated genes.^14^ Therefore, ARNT might be regulated directly or indirectly by HIF-1*α* in Hep3B cells.

Such a regulatory relationship between two transcription factors is a prerequisite of a feed-forward loop (FFL). FFLs have an important role in human signalling networks and cancer.^29, 30, 31^ According to the definition, a FFL is a network motif, which is composed of three elements. There are two input transcription factors, one of which regulates the other and both regulate a target gene in conjunction.^31, 32^ Elevation of ARNT by transient transfection increased reporter gene expression in normoxia and hypoxia. Noteworthily this effect can be mediated by functional HIF complexes of different composition including HIF-1*α*/ARNT heterodimers. In addition, these findings provide an explanation why such a FFL, that is, the capability to upregulate ARNT in hypoxia, might be an advantage for tumour cells. Increased ARNT expression was associated with an augmented HIF response, which implies that the amount of ARNT is rate limiting.

The counter intuitive increase in HRE-reporter activity after 20 min of re-oxygenation might be caused by the production of reactive oxygen species (ROS). It is well-known that re-oxygenation of hypoxic cells leads to elevated intracellular amounts of ROS such as superoxide.^33^ ROS generation in turn is able to activate the HIF pathway.^34^ Overexpression of ARNT further increased the reporter activity in re-oxygenated cells. Remarkably, the endogenous ARNT level decreased in a time-dependent manner when hypoxic Hep3B cells were returned to normoxic conditions. This observation is in line with a previous report published by Choi *et al.*^35^ Herein, the authors demonstrated that ARNT expression was linked to the intracellular redox state and attenuated in Hep3B cells owing to hydrogen peroxide treatment.^35^ Whether an elevated ARNT level provides a protection from cell damage caused by hypoxia/re-oxygenation events needs to be determined.

An increased expression of ARNT confers tumour cells a radioresistant phenotype (including Hep3B cells) and provides a clonal survival benefit as demonstrated previously by our group.^19^ The observation that ARNT overexpression in Hep3B cells is sufficient to stabilise HIF-1*α* accompanied with HRE-driven reporter gene expression in normoxia fits well with these results.^19^

The cellular capability to increase ARNT expression under hypoxic conditions might also improve the response to xenobiotics. In addition to its role in the HIF pathway, ARNT is a key player in the Aryl hydrocarbon receptor (AhR) signalling pathway, which is activated by chemical compounds.^16, 36^ An antagonistic crosstalk between AhR and HIF signalling via ARNT has been established but whether there is a competition for ARNT is unclear.^36^ Early evidence for such an antagonism was reported by *Gradin et al.*^37^ However, the results of our study support the concept of ARNT as being a limiting factor in HIF signalling. Whether an increased ARNT level facilitates xenobiotic responses in hypoxia needs to be evaluated.

Several studies have revealed the contribution of ARNT in tumour progression, drug- and radioresistance.^26, 38, 39^ Therefore, preventing this transcription factor from exerting its function was proposed as a treatment strategy in cancer therapy.^19, 39^ For instance, this can be accompanied by the inhibition of HIF-*α*/ARNT heterodimerisation by the drug Acriflavine.^2, 40^ Recently, a similar approach has been described by *Guo et al.*^8^ Herein, an artificial small molecule inhibitor which targets a specific domain of ARNT was used in order to prevent cofactor interactions.^8^ Theoretically, a facilitated degradation of ARNT might also be a future therapeutic approach. However, the turnover of ARNT is less investigated. There is evidence including from our own work that ARNT is not degraded by the proteasome under physiological conditions.^19, 35^ Nevertheless, it was proposed that proteasomal degradation of ARNT might occur as a stress response.^35^

**Figure 9:**
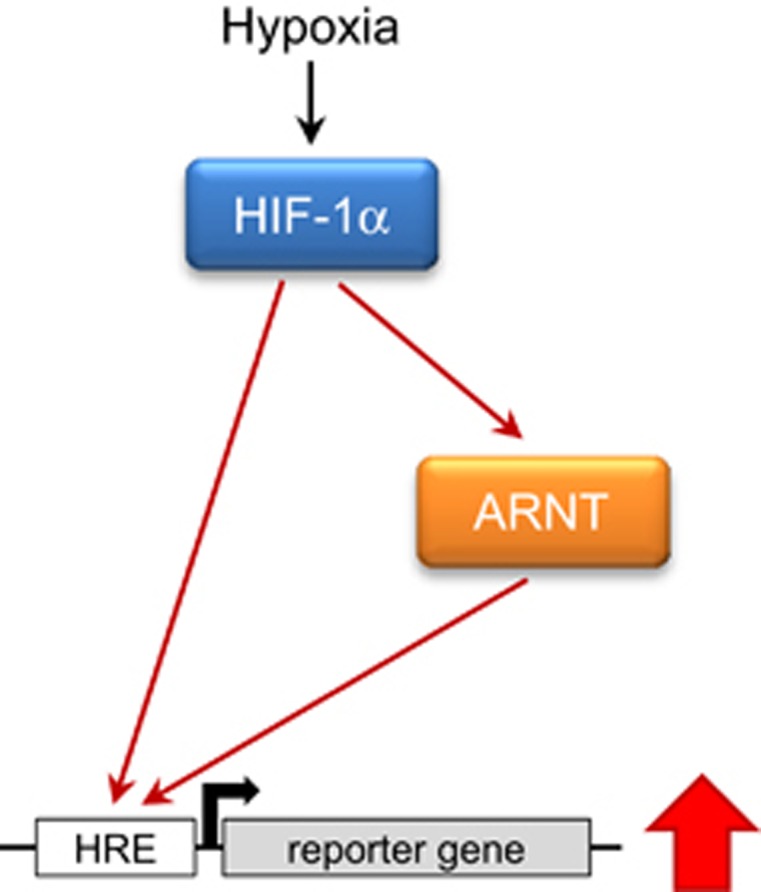
Feed-forward loop.

In conclusion, our results reveal a HIF-1*α*-dependent FFL, which amplifies HIF signalling in terms of elevated reporter gene expression by upregulation of ARNT (depicted in Figure 9). Undoubtedly, the presence of such a network motif adds a new layer of complexity to HIF biology. Our findings indicate that ARNT is a limiting factor regarding the HIF pathway in Hep3B cells. Therefore, augmented HIF signalling might provide a cellular benefit during tumourigenesis. Further studies are needed in order to elucidate genomic and/or epigenetic alterations mandatory to acquire this cellular capability. This might lead to the identification of tumours prone to HIF inhibition.

## Materials and Methods

### Chemicals

The protein synthesis inhibitor CHX (Sigma-Aldrich, Taufkirchen, Germany) was dissolved in dimethyl sulfoxide (DMSO, Sigma-Aldrich) with a stock concentration of 100 mmol/l, aliquoted and stored at −20 °C. Act D (Sigma-Aldrich), an inhibitor of RNA synthesis, was dissolved in DMSO (2 mg/ml) and stored in aliquots at −20 °C.

### Cell culture

Human hepatocellular carcinoma Hep3B cells (ATCC) were maintained in RPMI 1640 medium (Gibco, ThermoFisher Scientific, Waltham, MA, USA) containing 10% fetal bovine serum (FBS, Gibco) and penicillin/streptomycin. Cells were cultured at 37 °C in a humidified atmosphere with 5% v/v CO_2_. Hep3B cells were harvested by trypsinization and passaged 2–3 times weekly in a ratio of 1:5.

### Hypoxic conditions

Hep3B cells were counted using an automated trypan blue-exclusion technique (Cellometer, Nexcelom Bioscience, Lawrence, MA, USA) and seeded in appropriate culture dishes at a density of 3–4 × 10^4^ cells/cm^2^ following by an overnight incubation. On the next day, the supernatant was either replaced by fresh growth medium or the treatment required. Subsequently cells were exposed to hypoxia (3% v/v O_2_, 5% v/v CO_2_, balanced N_2_) for 8h or maintained under normoxic conditions.

### Gene silencing

Knockdown of HIF subunits was achieved by using a reverse siRNA transfection procedure performed in six-well plates. Therefore, for each well to be transfected 9 *μ*l Lipofectamine RNAiMAX (Life technologies, ThermoFisher Scientific) were mixed with 150 *μ*l Opti-MEM (Gibco) and combined with 200 pmol siRNA (BLOCK-iT Fluorescent Control siRNA, #442926, Life technologies; siHIF-1*α*, #L004018-00, Dharmacon, GE Healthcare, Lafayette, CO, USA; siHIF-2*α*, #4390825, Ambion, ThermoFisher Scientific) diluted in 150*μ*l Opti-MEM (Gibco). The transfection mixture was incubated at room temperature and cells were harvested in RPMI 1640 supplemented with 10% FBS without antibiotics. In total, 4 × 10^5^ Hep3B cells/well were mixed with the transfection mixture followed by the addition of 1ml antibiotic-free growth medium and incubated overnight. On the next day, the supernatant was replaced by 2 ml fresh antibiotic-free growth medium and cells were exposed to normoxia or hypoxia for 8 h.

### Overexpression studies

In total, 2 × 10^5^ Hep3B cells/well were seeded in six-well plates using antibiotic-free growth medium one day before transfection. Transient transfection of plasmids (pcDNA3, pcDNA3-HIF-1*α* (pHIF-1*α*), pcDNA3-ARNT (pARNT)) was carried out using GeneJuice (Merck Millipore, Darmstadt, Germany) according to the manufacturer's guidelines.

### Gene expression analysis

Gene expression was analysed as described previously.^19^ Concisely, mRNA expression was measured using TaqMan Gene Expression Assays (ARNT #Hs01121918_m1, HIF1A #Hs00936368_m1, Applied Biosystems, ThermoFisher Scientific) and matched to endogenous Beta-2-microglobulin (B2M) mRNA (#Hs00984230_m1, Applied Biosystems). Real-time PCR was performed with an ABI PRISM 7000 instrument (Applied Biosystems) using the ΔΔC_t_ method.

### Western blot analysis

Lysis of Hep3B cells was achieved using urea buffer as described previously.^19^ In brief, whole cell extracts containing 20 *μ*g or 50 *μ*g protein per lane (depending on type/purpose of experiment) were dissolved on a 7.5% acrylamide gel and blotted semi-dry onto a Polyvinyl difluoride (PVDF) membrane (Immobilon-P, 0.45 *μ*m, Merck Millipore). Membranes were blocked with 5% non-fat dry milk/PBS for 1h at room temperature and probed with specific antibodies (anti-HIF-1*α*: 1:1000, clone 54/HIF-1*α*, #610959, BD Transduction Laboratories, BD Biosciences, Heidelberg, Germany; anti-HIF-2*α*: 1:1000, polyclonal, #NB100-122, Novus Biologicals (R&D Systems Europe, London, UK); anti-ARNT: 1:2000, clone 2B10, #NB300-525, Novus Biologicals; anti-ARNT2: 1:1000, polyclonal, #sc-5581, Santa Cruz Biotechnology, Dallas, TX, USA) overnight at 4 °C. To verify equal loading of samples membranes were incubated with an anti-Actin (1:2000, polyclonal, #sc-1615, Santa Cruz), anti-*α*-Tubulin (1:1000, clone B-7, #sc-5286, Santa Cruz) or anti-Lamin A/C (1:1000, polyclonal, #sc-6215, Santa Cruz) antibody for 1h at room temperature. Detection was achieved by using appropriate HRP-conjugated secondary antibodies (1:5000, DAKO, Hamburg, Germany) in conjunction with the ECL reagent (Clarity Western ECL, Bio-Rad Laboratories, Munich, Germany). Afterwards membranes were exposed to X-ray films (Amersham Hyperfilm MP, GE Healthcare, Little Chalfont, UK). Quantitation of signals was performed using the AIDA Image Analyzer (Version 4.27, raytest).

### Reporter gene assays

In total, 4 × 10^4^ cells/well were seeded in 24-well plates using growth medium without antibiotics and allowed to adhere overnight. For transfection, the reporter plasmids HRE-*fLuc*, which encodes hypoxia-responsive *Firefly* luciferase and SV40-*rLuc*, encoding a constitutively expressed *Renilla* luciferase were mixed in Opti-MEM (Gibco) at equal amounts (100 ng/well to be transfected). The empty control plasmid (pcDNA3) and the ARNT expression vector (pcDNA3-ARNT) were diluted in Opti-MEM (Gibco), respectively (500 ng/well to be transfected) and combined with identical quantities of diluted reporter constructs. GeneJuice (Merck Millipore) transfection reagent (3 *μ*l/well to be transfected) was diluted in Opti-MEM (Gibco), mixed with plasmids and incubated at room temperature for 15 min. Meanwhile, the cell culture medium was renewed and the transfection mixture was added overnight. On the next day, the supernatant was replaced by 1ml fresh growth medium without antibiotics and cells were exposed to normoxia or hypoxia as indicated. All conditions were assayed in three technical replicates. Luciferase expression was determined using the Dual-Luciferase Reporter Assay System (#E1960, Promega, Fitchburg, WI, USA) according to the supplier's protocol. For time-course experiments the Dual-Glo Luciferase Assay System (#E2940, Promega) was used as described in the manufacturer's instructions. The luminescence signal was measured using an appropriate plate reader (Mithras LB940, Berthold Technologies, Bad Wildbad, Germany). *Firefly/Renilla* luciferase ratios were calculated and normalised to vector-transfected control cells.

### Statistical analysis

GraphPad Prism 4 software (GraphPad) was used in order to perform statistical analysis of experimental results. All values are given as mean±S.E.M. Each experiment was conducted independently at least three times. Analysis of normalised data of only two groups was done using the one-sample *t*-test. Comparison between two groups was achieved using the unpaired *t*-test. *P*-values ⩽0.05 were considered as statistically significant.

## Abbreviations

Act D: actinomycin D
ARNT: aryl hydrocarbon receptor nuclear translocator
CHX: cycloheximide
DMSO: dimethyl sulfoxide
FBS: fetal bovine serum
FFL: feed-forward loop
HCC: hepatocellular carcinoma
HIF: hypoxia-inducible factor
HRE: hypoxia-responsive element
PHD: prolyl hydroxylase domain enzymes
PVDF: polyvinyl difluoride
pVHL: von Hippel-Lindau tumour suppressor protein
qRT-PCR: quantitative reverse transcription-polymerase chain reaction
ROS: reactive oxygen species
S.E.M.: standard error of the mean
