# Supplementary figures and images for: A HIF-1α-driven feed-forward loop augments HIF signalling in Hep3B cells by upregulation of ARNT
Source: Cell Death Dis. 2016 Jun 30;7(6):e2284–. doi: 10.1038/cddis.2016.187 (PMC5108338; doi:10.1038/cddis.2016.187)

## Slide 1
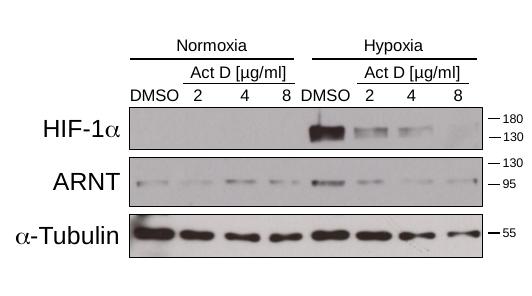

Supplement: Supplementary Figure S1 [file cddis2016187x1.ppt]
