# Supplementary material for: A HIF-1α-driven feed-forward loop augments HIF signalling in Hep3B cells by upregulation of ARNT
Source: Cell Death Dis. 2016 Jun 30;7(6):e2284–. doi: 10.1038/cddis.2016.187 (PMC5108338; doi:10.1038/cddis.2016.187)
Supplement: Supplementary Figure Legend [file cddis2016187x2.doc]

**Figure S1:** Treatment of Hep3B cells with Actinomycin D (Act D). Cells were incubated with increasing concentrations of Act D in normoxia (N) and hypoxia (H, 3% O_2_) for 8h as indicated. DMSO at the highest concentration (0.4 v/v %) was used as vehicle control. HIF-1α and ARNT protein levels were determined by Western blot analysis. Representative result of n=3 independent experiments.
